# Deep Learning-Based Multi-Omics Data Integration Reveals Two Prognostic Subtypes in High-Risk Neuroblastoma

**DOI:** 10.3389/fgene.2018.00477

**Published:** 2018-10-18

**Authors:** Li Zhang, Chenkai Lv, Yaqiong Jin, Ganqi Cheng, Yibao Fu, Dongsheng Yuan, Yiran Tao, Yongli Guo, Xin Ni, Tieliu Shi

**Affiliations:** ^1^Center for Bioinformatics and Computational Biology, and the Institute of Biomedical Sciences, School of Life Sciences, East China Normal University, Shanghai, China; ^2^Beijing Key Laboratory for Pediatric Diseases of Otolaryngology, Head and Neck Surgery, MOE Key Laboratory of Major Diseases in Children, Beijing Children's Hospital, National Center for Children's Health, Beijing Pediatric Research Institute, Capital Medical University, Beijing, China; ^3^Biobank for Clinical Data and Samples in Pediatrics, Beijing Children's Hospital, National Center for Children's Health, Beijing Pediatric Research Institute, Capital Medical University, Beijing, China; ^4^Department of Otolaryngology, Head and Neck Surgery, Beijing Children's Hospital, National Center for Children's Health, Capital Medical University, Beijing, China

**Keywords:** deep learning, high-risk neuroblastoma, multi-omics data integration, *MYCN* amplification, machine learning

## Abstract

High-risk neuroblastoma is a very aggressive disease, with excessive tumor growth and poor outcomes. A proper stratification of the high-risk patients by prognostic outcome is important for treatment. However, there is still a lack of survival stratification for the high-risk neuroblastoma. To fill the gap, we adopt a deep learning algorithm, Autoencoder, to integrate multi-omics data, and combine it with K-means clustering to identify two subtypes with significant survival differences. By comparing the Autoencoder with PCA, iCluster, and DGscore about the classification based on multi-omics data integration, Autoencoder-based classification outperforms the alternative approaches. Furthermore, we also validated the classification in two independent datasets by training machine-learning classification models, and confirmed its robustness. Functional analysis revealed that *MYCN* amplification was more frequently occurred in the ultra-high-risk subtype, in accordance with the overexpression of *MYC/MYCN* targets in this subtype. In summary, prognostic subtypes identified by deep learning-based multi-omics integration could not only improve our understanding of molecular mechanism, but also help the clinicians make decisions.

## Introduction

Neuroblastoma is the most common extracranial solid tumor in childhood (mostly under the age of five) and accounts for approximately 15% of childhood cancer mortality (Ward et al., 2014). It can develop anywhere in the sympathetic nervous system (Maris et al., 2007). Sixty percent of the tumors occur within the abdomen, commonly in the adrenal medulla. The clinical hallmark of neuroblastoma is heterogeneity, with the outcomes of tumor progression varying widely. According to the Children's Oncology Group (COG) assignment, age at diagnosis, the stage of disease, *MYCN* amplification (Brodeur et al., 1984; Tomioka et al., 2008), the International Neuroblastoma Pathology Classification and DNA ploidy are employed to stratify risk groups. Low-risk group has good outcome, whereas high-risk disease presents poor outcome even with the most intensive multi-modal therapies. Several recurrently mutated genes or loci which correlated with high-risk neuroblastoma have been identified, such as *ALK* (Mosse et al., 2008) mutations or amplifications, *PHOX2B* (Brodeur et al., 1984) mutation, chromosome 1p and 11q deletions, truncating or structural variants of *ATRX* gene (Cheung et al., 2012; Molenaar et al., 2012), genomic rearrangements of TERT (Peifer et al., 2015; Valentijn et al., 2015). These genetic events cover 92% of high-risk neuroblastoma (Peifer et al., 2015).

Driver genes/alterations, such as *MYCN*, 1p/11q deletion, *ALK, ATRX* and *TERT*, are characterized in high-risk neuroblastoma by previous studies, however, it is difficult to further stratify the high-risk neuroblastoma at molecular level. Previous studies have mostly intended to predict high-risk neuroblastoma survival using only genomic alterations (Stigliani et al., 2012) or dysregulated genes (Blanc et al., 2005; Chen et al., 2016; Wei et al., 2018), rarely by multi-omics integration. Therefore, the lack of prognostic stratification for high-risk neuroblastoma by multi-omics data integration motivated us to conduct this study.

With the production of omics data, such as The Cancer Genome Atlas (TCGA) and Therapeutically Applicable Research to Generate Effective Treatments (TARGET) projects, multi-omics integration is much needed in cancer researchers. Recently, Suo et al. (2018) have proposed a driver-gene score (DGscore) approach to predict the prognosis of the high-risk neuroblastoma by integrating the genome and transcriptome data. However, small sample size and no independent data for validation are the major limitations. Moreover, integrative clustering (iCluster) analysis (Shen et al., 2009; Cancer Genome Atlas Research, 2014) and PCA-based clustering analysis (Alexe et al., 2007; Nicolau et al., 2011) are widely applied to cancer subtyping. iCluster analysis could not only identify the molecular subtypes, but also associate the multi-omics data with each other. PCA is able to reduce the dimensionality of the multi-omics data, and integrates high dimensional multi-omics data into principal components. In addition, deep learning-based algorithm has been proposed to identify cancer subtypes. The recent study (Chaudhary et al., 2018) using deep learning-based multi-omics data integration robustly predicts survival in liver cancer. However, the multi-omics data integration approaches are rarely applied to neuroblastoma subtyping.

In this study, we used multi-omics-based unsupervised learning to stratify the high-risk neuroblastoma based on the new features re-encoded by Autoencoder algorithm, and compared the stratification with those identified by iCluster or PCA. The stratification of high-risk neuroblastoma by Autoencoder was also validated in two independent datasets, which may not only help the clinicians make rational and efficacious chemotherapeutic protocols, but also demonstrate that the deep learning-based algorithm is very efficient in multi-omics integration.

## Materials and methods

### Datasets and study design

We used multi-omics data from two projects in this study: Therapeutically Applicable Research to Generate Effective Treatments (TARGET) project (Pugh et al., 2013) and Sequencing Quality Control (SEQC) project (Zhang et al., 2015). The TARGET cohort is comprised of 407 high-risk neuroblastoma samples, including 217 samples with gene expression data and 380 samples with copy number alterations (CNA). Among these obtained samples, 190 has both gene expression and CNA data. The SEQC cohort has a total of 498 neuroblastoma samples, including 176 high-risk and 322 low- or intermediate-risk samples. The survival data were publicly available at the official website of TARGET project (https://ocg.cancer.gov/programs/target/data-matrix), and GEO database with accession number GSE49711 for SEQC cohort.

To integrate gene expression and CNA data, we first stacked these two datasets by the190 overlapping samples from TARGET cohort to form a new one. Then we selected the initial prognostic features (genes or CNAs) with Cox regression (log rank test, *P* < 0.05) for further analysis.

This new dataset with selected initial prognostic features was used in these following parts of our work: generating new features from a classic artificial neural network: Autoencoder (with which 100 new features were generated and Cox regression was applied again here to ensure that they were significantly prognostic), obtaining labels for different survival-risk groups through K-means clustering from transformed new features by Autoencoder, and training classifiers with models such as SVM, Naïve Bayes, and logistic regression according to the class labels.

Also, to demonstrate the robustness of the classification at predicting prognosis, these supervised classification methods mentioned above, along with XGBoost, were trained on gene expression data and CNA data respectively by different machine learning methods.

There were two datasets used for demonstrating the robustness of the classification for predicting prognosis, one was the remaining 190 samples which had CNA data only (the internal validation set), and the other was 176 high-risk samples with gene expression data in SEQC project (the external validation set). The class labels for samples from TARGET internal validation set and SEQC external validation set were predicted by CNA-based XGBoost and gene expression-based SVM models, respectively.

### Gene expression data from target and SEQC projects

The gene expression data from TARGET project were profiled by Affymetrix Exon ST platform, and normalized by Robust Multi-array Average (RMA) procedure, which could be downloaded from the website (https://ocg.cancer.gov/programs/target/data-matrix).

As reported by Zhang et al. (2015), a total of 498 neuroblastoma samples were selected for RNA sequencing, of which, 176 high-risk neuroblastoma cases were selected for external validation. The RNA sequencing reads of 176 high-risk neuroblastoma samples were mapped to human reference genome GRCh37/hg19 with GENCODE gene annotation v19 by hisat2. The gene expression were then quantified by StringTie (Pertea et al., 2015) with default options and combined in R programming software with *ballgown* package.

### Data integration and re-coding by autoencoder

Autoencoder is a dimensionality reduction method based on artificial neural network, which consists of input, hidden, and output layers. The data integration analysis by Autoencoder was implemented in R programming software with package *ANN2*. To better capture properties that reflect the variation of patients' prognosis, a classic autoencoder with 3 hidden layers was applied (500, 100, and 500 nodes, respectively), of which the 100-node bottleneck layer was used to represent new features for further analysis. We then selected 35 survival-associated features (log-rank test, *P*-value < 0.05) from the 100 new features.

For a given layer, a specific activation function was assigned, and the output x' was given by a composite function of x, which was composed of all these activation functions from each layer, and could be expressed as:

$$\begin{array}{l} \;\gamma =f_{i}\left(x\right)=\tanh\left(W_{i.}x+b_{i}\right)\\ x^{\prime }=F_{1\to k}\left(x\right)=f_{1}\cdots f_{k-1}f_{k}\left(x\right), \end{array}$$

where k represents to the number of layers.

We measured the error with function *the Pseudo-Huber loss function*, which ensures that derivatives are continuous for all degrees, that is:

$$L\left(x,x^{\prime }\right)=\sum_{k=1}^{n}\left[\delta^{2}\sqrt{1+\left(\frac{x_{k}-x_{k}^{\prime }}{\delta }\right)2-1}\right]$$

where n stands for the dimension of the input data. As can be seen in Supplementary Figure S1, the output data from reconstructed layer was compared with the raw input data with Pseudo-Huber loss function.

The Autoencoder was trained using the gradient descent algorithm with 10 epochs, a batch size of 32, and a learning rate of 1e-6. The parameters of L1 and L2 regularization were set to 0.0001 and 0.001.

### Gene expression data normalization

For the RMA-based gene expression data by microarray platform, we transformed the expression value as Z-score for each gene. For the gene expression data of RNA-seq, we firstly calculated the fractions of the genes that had a FPKM value over the threshold we set, which can be seen in the Supplementary Table S5 (We selected several possible thresholds, e.g., 0.01, 0.05, 0.1, 0.5, 1). We then applied an alternative method, instead of adding an arbitrary value, we calculated the minimal value for each sample which is not zero, and then set all values below maximum of these minimums (which is 3.7e-05) to the minimum of these minimums (which is 1.60e-07) in all samples, and then transformed by logarithm with base-2. Like the gene expression data by microarray platform, the gene expression values were also transformed to Z-scores in similar manner.

### CNA data annotation

The segmented copy number regions with segment means were available at the TARGET website (https://ocg.cancer.gov/programs/target/data-matrix). We merged the segmented CNAs from the 380 samples, and annotated the genes in the CNAs by GISTIC2.0 (Mermel et al., 2011), which is implemented in GenePattern (Reich et al., 2006), a webserver publicly available for researchers (https://software.broadinstitute.org/cancer/software/genepattern/). The rows and columns of the CNA matrix represent the genes and samples, respectively. Each element of the CNA matrix was normalized as log_2_ (segmented copy number)−1.

### Feature and model selection

To integrate the multi-omics data, we applied three methods: autoencoder-based deep learning, iCluster and PCA, and then we compared the labels identified by these three approaches. Unlike iCluster, autoencoder-based deep learning and PCA were not clustering algorithms, thus the other two were followed by k-means clustering. Taking together, these three methods were able to integrate multi-omics data and were evaluated by the association between classification labels and patients' prognosis.

Machine learning classifiers such as SVM, Naïve Bayes, and logistic regression are supervised learning algorithms. The classification labels used for these machine learning classifiers were only determined by autoencoder-based deep learning followed by K-means clustering, not by the other methods. After obtaining the labels from K-means clustering, we need to examine the robustness of this sample stratification. We then built two supervised models based on the gene expression and CNA data, respectively, and predicted the classes for samples from both internal and external validation sets. The machine-learning classification models were then used to test its robustness in validation sets.

Features for the models, including Naïve Bayes, logistic regression and SVM, were selected by a backward elimination manner. For each gene or CNA, the importance was evaluated by the ANOVA *F*-value. 10-fold cross-validation with 10-time repeat was conducted to evaluate the predictive ability of the selected features (genes or CNAs). The feature combinations with highest average predictive accuracy were selected. The features for XGBoost were selected by its internal algorithm. Given the features, receiver operating characteristic (ROC) curve was plotted for each model, and the one with highest area under the curve (AUC) was selected as the prediction model.

### Statistical analysis

The statistical analyzing methods such as Cox proportional hazards (Cox-PH) analysis, principal component analysis, K-means clustering, integrative clustering and student-*t* test were implemented in R programming software with version 3.5.0. In addition, we determined the optimal number of clusters on three metrics: C index for the prognostic differences, Silhouette index and Calinski–Harabasz criterion (Supplementary Table S4). The overrepresentation enrichment analysis (OEA) was implemented in WebGestalt (Wang et al., 2017) (http://www.webgestalt.org/option.php) with a functional database named Hallmark50 (Liberzon et al., 2015).

## Results

### Data collection and pre-processing for integrative analysis

We collected 407 high-risk neuroblastoma samples from TARGET project (Ma et al., 2018), including 217 samples with gene expression data and 380 samples with copy number alterations (CNA). The neuroblastoma patients were treated according to Children's Oncology Group (COG) risk-group assignment. Among the obtained tumor samples, 190 had both gene expression and CNA data, which were used as training data in this study. Multi-omics data in the training data were integrated to discover a prognostic stratification of the high-risk neuroblastoma. The remaining 190 samples with only CNA data were used as an internal validation data to test the robustness of classification. In addition, we also collected RNA sequencing data of 176 high-risk neuroblastoma samples from SEQC project, which was used as an external validation data to further test the robustness.

**Figure 1:**
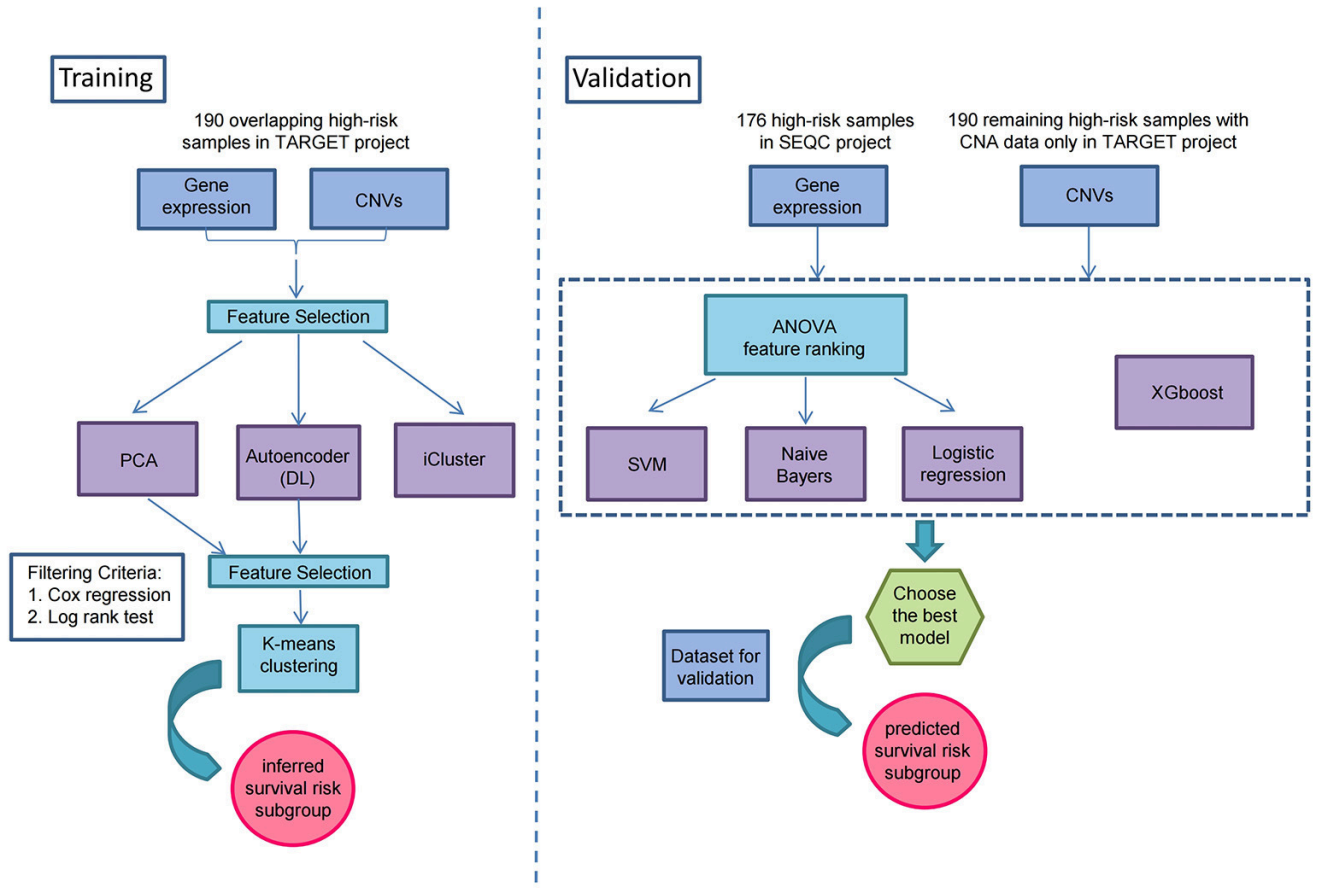
Overview workflow for the identification of prognostic subtypes by Autoencoder-based multi-omics data integration in high-risk neuroblastoma.

As illustrated in Figure 1, prior to multi-omics integration, prognosis-associated genes were selected from both gene expression and CNA data of the 190 NB samples based on the univariate Cox proportional hazards (Cox-PH) regression analysis. Finally, 2,218 aberrantly expressed genes and 497 copy number altered genes were associated with the prognosis of high-risk neuroblastoma [*P*-value < 0.05 for event-free survival (EFS) or overall survival (OS)], which were used for integrative analysis later on.

### Identification of prognostic subtypes in high-risk neuroblastoma

To identify the prognostic subtypes in high-risk neuroblastoma, we stacked the two matrices of gene expression and CNA by the 190 overlapping samples in TARGET project, and transformed the initial prognostic features into 100 new features according to Autoencoder, a five-layer neural network with three hidden layers (500, 100, and 500 nodes). The two-omics data were integrated and represented by the 100 new features obtained from the bottleneck layer of the autoencoder. We then conducted a univariate Cox-PH regression on each of the 100 new features and identified 35 features significantly (*P* < 0.05) associated with EFS or OS. Subsequently, K-means clustering analysis was performed on the 35 new features with clustering number ranging from 2 to 6 (Figure 1). We determined the optimal number of clusters based on three metrics: C index for the prognostic differences, Silhouette index and Calinski–Harabasz criterion, which consistently supported our choice of 2 as the number of clusters (Supplementary Table S4). Finally, we clustered the samples into two subtypes, which were defined as G1 and G2.

**Figure 2:**
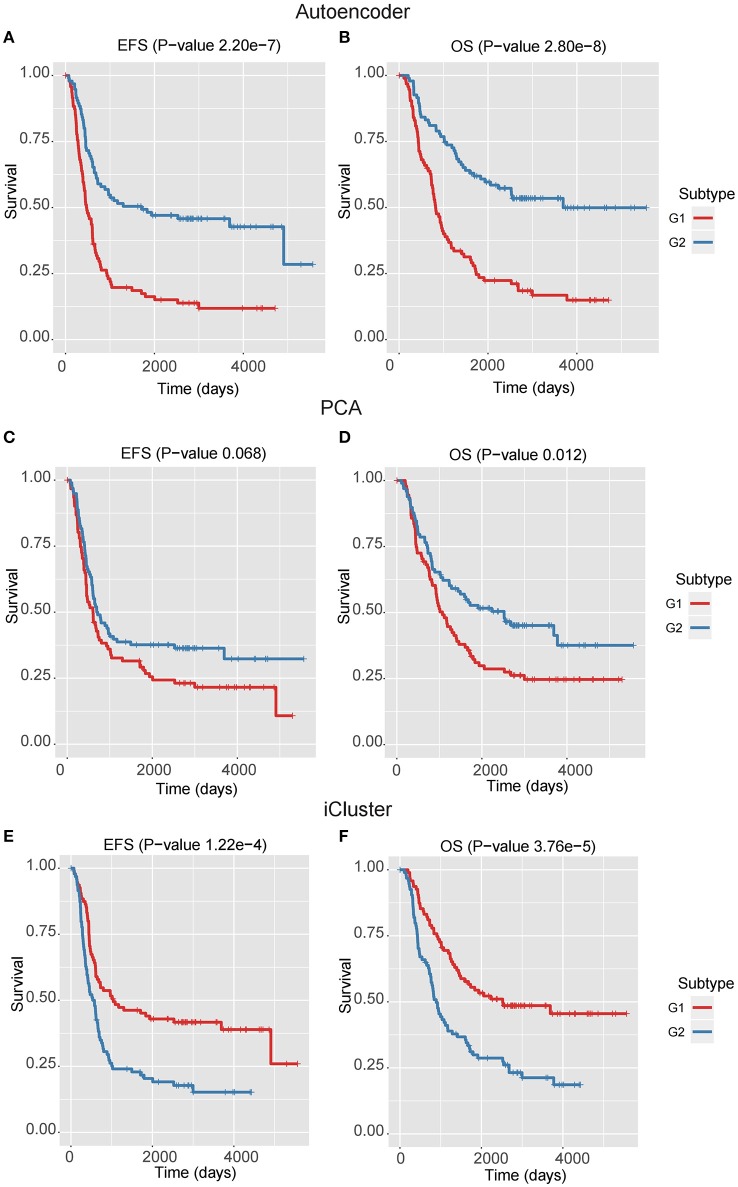
The Kaplan–Meier curves for EFS or OS of two identified subtypes by three multi-omics integration algorithms, Autoencoder **(A,B)**, PCA **(C,D)**, and iCluster **(E,F)**.

We next assessed the prognostic difference between these two subgroups by univariate Cox-PH regression, and observed that the G1 exhibited worse prognosis than G2 (*P*-value < 0.0001 for both EFS and OS, Figures 2A,B), indicating that G1 was an ultra-high-risk subtype. Moreover, the concordance index (C-index), which measures the fraction of all pairs of cases whose predicted survival times are ordered correctly, was also calculated. Expectedly, our classification also generated high C-index (0.74 ± 0.08 for EFS and 0.71 ± 0.08 for OS). The result indicated that our classification revealed two prognostic subtypes in high-risk neuroblastoma.

### Autoencoder-based multi-omics integration outperforms alternative approaches

In addition to Autoencoder-based multi-omics integration, principal component analysis (PCA) and integrative clustering analysis (iCluster) were also incorporated to evaluate the performance of multi-omics integration approaches (Supplementary Table S1). Similar to the 100 new features by Autoencoder, PCA transformed the inital features into 100 principal components, and Cox-PH was applied to select prognostic principal components. As a result, 14 principal components were remained. Unlike PCA and Autoencoder, the iCluster analysis did not have to transform the initial prognostic features into new features, but placed cases into groups based on both gene expression patterns and copy number status.

In the training data, we found that the classification by Autoencoder had better performance than the other two approaches (Figures 2C–F), among which iCluster achieved high C-index and significant log-rank *P*-value, but it was still less significant as compared with the model using Autoencoder, and the PCA-based classification showed poor performance, especially failing to give significant log-rank *P* value for EFS (*P* = 0.068). In addition, as compared with the DGscore method (*P*-value = 0.006), Autoencoder-based classification also achieved higher statistical significance (*P* = 5.66e-6 for EFS and *P* = 1.28e-5 for OS). The result indicated that Autoencoder-based multi-omics integration outperformed these alternative approaches.

### Prognostic subtypes are validated in two validation datasets

To demonstrate the robustness of the classification at predicting prognosis, we built two supervised classification models based on gene expression and CNA data separately to predict the classification labels for samples from both internal and external validation datasets, respectively.

After obtaining the labels from K-means clustering, we first built two supervised models based on the gene expression and CNA data, respectively. Each omics data was normalized as Z-score to avoid platform differences. For the internal validation, we used the remaining 190 samples with only CNA data from the TARGET project which didn't overlap with the samples with gene expression data. Meanwhile, the 176 SEQC high-risk neuroblastoma samples with gene expression data was used as external validation.

**Figure 3:**
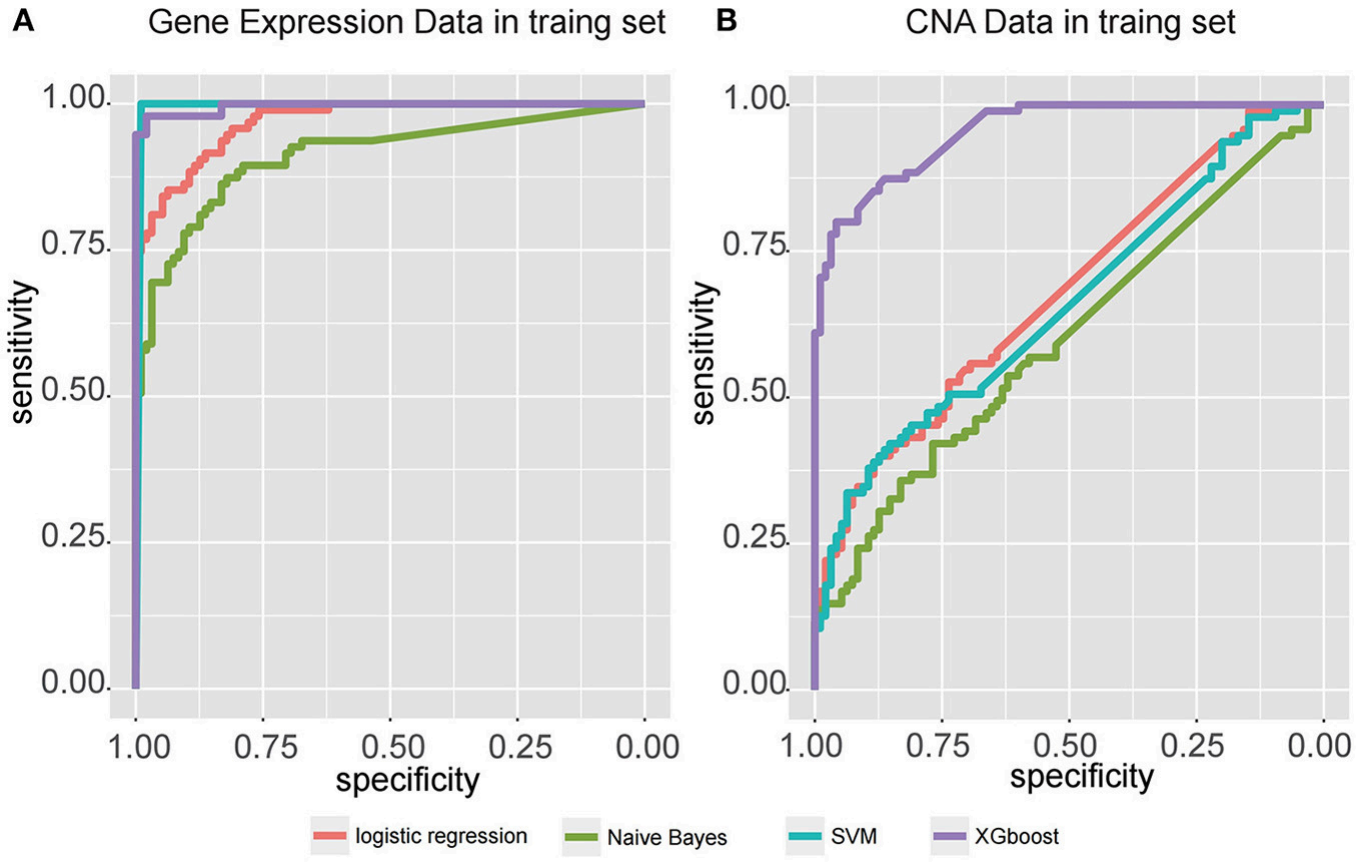
Receiver operating characteristic (ROC) curve for four classifiers, including logistic regression, Naïve Bayes, SVM, and XGBoost, that predict the subtypes of samples from two independent datasets, **(A)** gene expression data from SEQC external validation cohort, and **(B)** CNA data from TARGET internal validation cohort.

**Figure 4:**
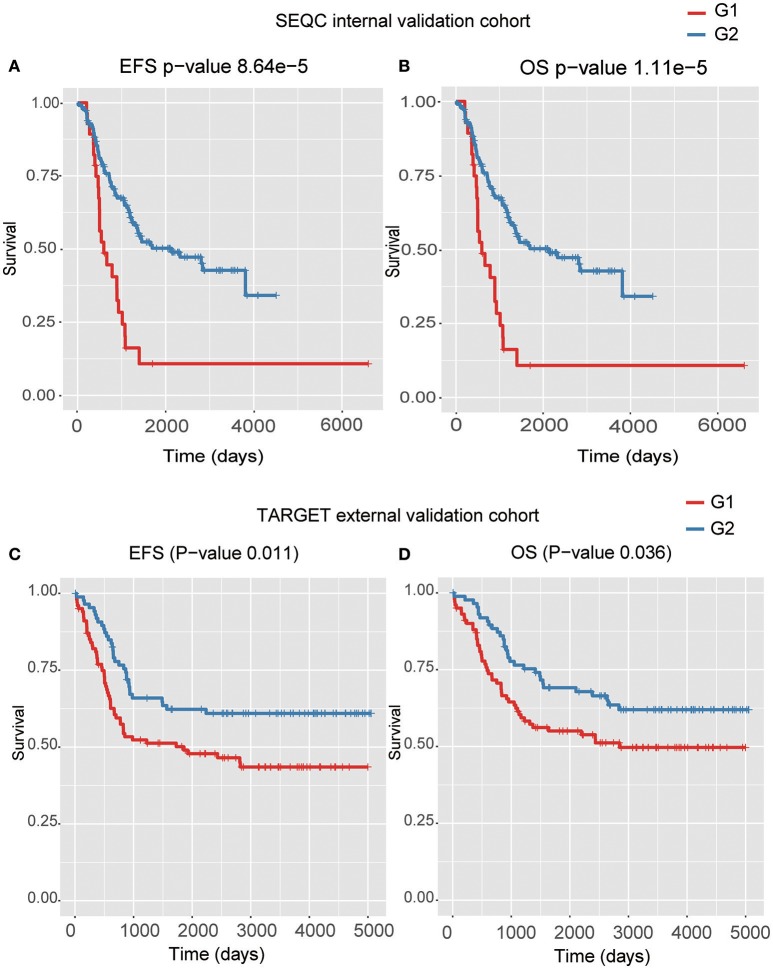
The Kaplan–Meier curves for EFS or OS of two predicted subtypes for the high-risk tumors from SEQC external validation cohort **(A,B)** and TARGET internal validation **(C,D)** cohort.

Four models, including SVM, naïve Bayes, logistic regression, and XGBoost, were built to select the best model for classification prediction. Based on ten-fold cross-validation in the training dataset, SVM exhibited high capability of predicting classification labels for 176 samples from the external validation set using gene expression data (See features in Supplementary Table S2), while XGBoost achieved higher performance on the CNA data than other models (Figure 3, Table 1, and see features in Supplementary Table S3). For the gene expression data from SEQC project, we achieved good C-indices (0.69 ± 0.08 for EFS and 0.74 ± 0.08 for OS) and log-rank *P* values (< 0.0001) between the two subtypes (Figures 4A,B). For the CNV data from TARGET internal validation cohort, the classification had C-indices over 0.64 and low log-rank *P* values (*P* < 0.05, Figures 4C,D). The validation of the classification in both internal and external datasets further demonstrated that the two subtypes indeed had different outcomes.

**Table 1:** Performance of four classifiers using the training dataset.

**Feature selection + classifier**	**Feature type**	**Feature number**	**AUC**	**Average accuracy**	**Average AUC**
ANOVA + SVM	GE	56	0.9962	0.7553	0.8446
	CNA	30	0.6586	0.5937	0.5159
ANOVA + naïve bayes	GE	46	0.9299	0.6755	0.8291
	CNA	24	0.6019	0.5234	0.5506
ANOVA + logistic regression	GE	44	0.9703	0.7059	0.6053
	CNA	15	0.6782	0.6135	0.5699
Xgboost	GE	64	0.9602	0.7338	0.8025
	CNA	30	0.954	0.6559	0.6317

*GE, gene expression; CAN, copy number alteration; ANOVA, analysis of variance; SVM, support vector machine; AUC, area under the curve; Average accuracy, average of the accuracies from 10-fold cross-validation. Average AUC, average of the AUC values from 10-fold cross-validation*.

### Functional analysis of the prognostic subtypes in high-risk NB

We used *t*-test for differential gene expression between the two subtypes of both training and validation datasets. At the FDR < 0.05 for both datasets, we obtained 302 upregulated and 851 downregulated genes in the subtype G1. Overrepresentation enrichment analysis (OEA) was then performed on the two gene sets (Table 2). We identified MYC target genes, including *FARSA, SLC29A2, PLK1, WDR74, RRP9*, and *IMP4*, were upregulated in G1 subtype (FDR < 0.05). Interestingly, *MYCN* amplification (MNA) was observed to present higher frequency in G1 than G2 (*P* = 0.054, 35 vs. 26% in training data, and *P* < 0.005, 77 vs. 44% in the validation data), indicating that our classification was associated with MNA to a certain extent, but some samples without MNA also had poor survival. However, we did not identify significant down-regulated pathways in G1 subtype. Alternatively, interferon-alpha response pathway was down-regulated in G1 (*P*-value < 0.05), which is a common defect in human cancers (Critchley-Thorne et al., 2009). In detail, the genes in interferon-alpha response pathway, such as CMTR1, NUB1, and STAT2, were consistently down-regulated in G1 subtype. The result indicated that interferon-alpha may be a potential immunotherapy strategy for the ultra-high risk neuroblastoma.

**Table 2:** Hallmark gene sets identified by OEA (FDR < 0.05).

**Status**	**Gene set**	**Description**	***P*-value**	**FDR**
Up	HALLMARK_MYC_TARGETS_V2	MYC targets, variant 2	9.81E-07	4.9E-05
Down	HALLMARK_INTERFERON_ALPHA_RESPONSE	Interferon-alpha response	5.14E-03	5.76E-01

## Discussion

Recently, with the development of high-throughput technologies, such as DNA microarray, next generation sequencing, and mass spectrum-based proteomics, huge amounts of omics data are produced and made available publicly. However, high production of multi-omics data also raises requirements to comprehensively analyze different levels of omics data.

In the present study, we have adopted a deep learning-based algorithm, Autoencoder, to integrate copy number alterations and gene expression data to identify two prognostic subtypes, defined as G1 and G2, in high-risk neuroblastoma. The subtype G1 exhibits worse prognosis than G2 in both EFS and OS (*P*-value < 0.0001). The Autoencoder-based classification also generates high C-index (0.74 ± 0.08 for EFS and 0.71 ± 0.08 for OS). The performance comparison of Autoencoder with PCA and iCluster demonstrates that our Autoencoder-based classification is superior to the two alternative approaches. Moreover, the result of Autoencoder-based classification is also more significant than DGscore method. To demonstrate the robustness of the classification, we build two supervised classifiers for the independent CNA and gene expression datasets, respectively. For both of the datasets, we achieve good C-indices and significant log-rank *P*-values (*P* < 0.05). We thus conclude that Autoencoder-based classification outperforms other approaches, and we speculate that the unique advantage of the Autoencoder, which can capture the core features relevant to the prognosis, have contributed to this.

High-risk neuroblastoma is an aggressive disease. To our knowledge, the present study is the first to apply deep learning approach to distinguish ultra-high-risk subgroup from the high-risk neuroblastoma, with validation in independent datasets. The integrative classification of the high-risk neuroblastoma may help clinicians develop personalized treatment programs, and better predict patients' prognosis.

## Conclusion

Prognostic subtypes identified by deep learning-based multi-omics integration could not only improve our understanding of molecular mechanism, but also help the clinicians make decisions.

## Author contributions

In this study, TS designed the study. LZ and CL adapted algorithms and software for building model and data analysis. LZ, CL, and YJ interpreted data in context of NB biology. LZ, YJ, CL, and GC drafted the manuscript. YF, DY, and YT collected the data. TS, XN, and YG revised and finalized the manuscript. All authors read and approved the final manuscript.

### Conflict of interest statement

The authors declare that the research was conducted in the absence of any commercial or financial relationships that could be construed as a potential conflict of interest.

## Supplementary material

Supplementary Figure S1 The schematic diagram for Autoencoder algorithm.

Supplementary Table S1 Prognostic subtypes identified by three algorithms.

Supplementary Table S2 Gene features used by SVM.

Supplementary Table S3 CNA genes selected by XGBoost.

Supplementary Table S4 Scores of different cluster numbers.

Supplementary Table S5 Percentages of genes among different thresholds based on expression.
