# Supplementary figures and images for: Deep Learning-Based Multi-Omics Data Integration Reveals Two Prognostic Subtypes in High-Risk Neuroblastoma
Source: Front Genet. 2018 Oct 18;9:477. doi: 10.3389/fgene.2018.00477 (PMC6201709; doi:10.3389/fgene.2018.00477)

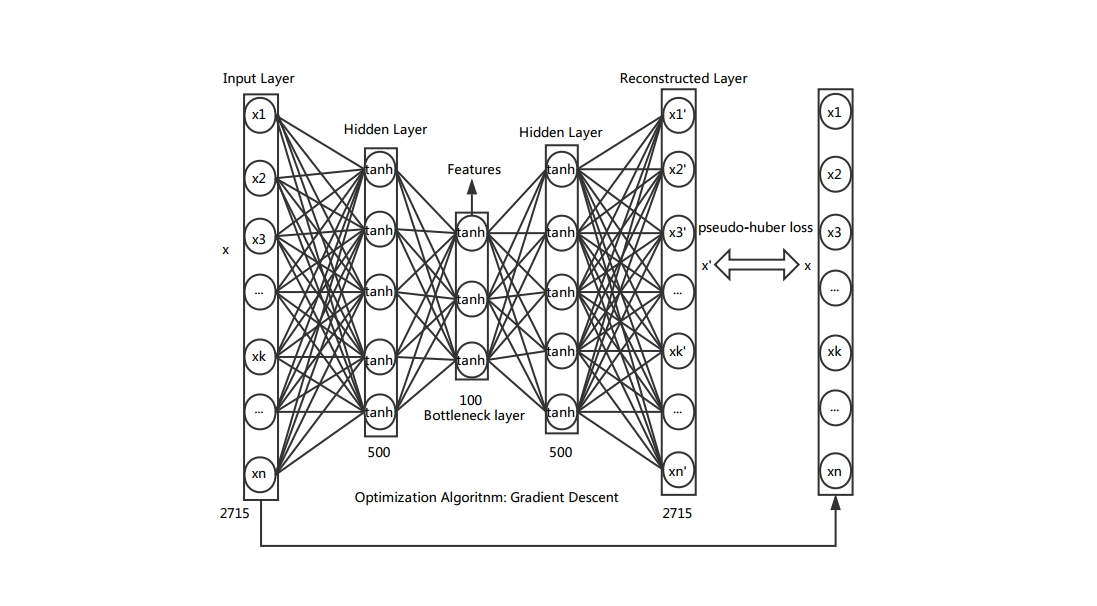

Supplement: Supplementary Figure S1 — The schematic diagram for Autoencoder algorithm. [file Image_1.tiff]
